# Malakoplakia as a cause of severe hypercalcemia through ectopic 25-hydroxyvitamin D3 1-alpha-hydroxylase expression: A case report

**DOI:** 10.1097/MD.0000000000012090

**Published:** 2018-10-05

**Authors:** Jonathan Maurice Chemouny, Aurélie Sannier, Guillaume Hanouna, Laure Champion, Francois Vrtovsnik, Eric Daugas

**Affiliations:** a Service de Néphrologie, Hôpital Bichat-Claude Bernard, AP-HP, DHU Fire; b INSERM, Centre de Recherche sur l’Inflammation; c Université Paris Diderot, Sorbonne Paris Cité; d Laboratoire d’anatomopathologie et de cytologie, Hôpital Bichat-Claude Bernard, AP-HP, Paris, France.

**Keywords:** calcitriol, hypercalcemia, malakoplakia

## Abstract

**Rationale:**

Malakoplakia is a rare disease characterized by the presence of nongranulomatous macrophage infiltration. In most cases, it affects the urinary tract. Malakoplakia can cause acute kidney injury when it is localized in the kidneys.

**Patient concerns:**

Here, we report the case of a 65-year-old female patient with renal malakoplakia responsible for hypercalcemia. During her initial assessment, she was also diagnosed 25-OH vitamin D insufficiency, for which she was prescribed oral cholecalciferol. Three months later, she developed severe hypercalcemia with normal 25-OH vitamin D and parathyroid hormone levels and high 1,25-dihydroxyvitamin D levels.

**Diagnoses:**

After a superimposed granulomatous disease was excluded, malakoplakia cells were suspected to be responsible for the abnormal 25-hydroxyvitamin D3 1-alpha-hydroxylase activity, which was confirmed by immunohistochemistry.

**Interventions:**

Cholecalciferol was stopped, the patient was rehydrated with intravenous physiological saline, and prednisone was initiated to decrease the enzyme activity.

**Outcomes:**

Six months later, she displayed normal serum calcium, 25-OH vitamin D and 1,25-dihydroxyvitamin D levels.

**Lessons:**

This case illustrates that malakoplakia may exhibit ectopic 25-hydroxyvitamin D3 1-alpha-hydroxylase activity and cause severe hypercalcemia upon vitamin D supplementation. Therefore, such supplementation should not be given in malakoplakia patients without an actual deficiency and requires careful monitoring of serum calcium.

## 1 Introduction

Malakoplakia is a rare disease characterized by the presence of nongranulomatous macrophage infiltration located in the urinary tract in most cases,^[1]^ although localizations outside the urinary tract, especially in the gastrointestinal system, have been described.^[2]^ It primarily affects women over 40 years old.^[3]^ Malakoplakia is characterized by an infiltration of macrophages with a granular eosinophilic cytoplasm (von Hansemann cells) containing Michaelis–Gutmann bodies.^[1]^ The clinical manifestations are usually direct consequences of anatomical and functional alterations of the affected organ and may be associated with signs of general inflammation, such as fever or weight loss. For instance, patients with renal parenchymal malakoplakia commonly present with fever, loin pain and enlarged kidneys with inconstant renal dysfunction.^[4–6]^ We report here^[7]^ a new systemic manifestation of malakoplakia. In this patient, malakoplakia was indeed responsible for severe hypercalcemia through ectopic expression of 25-hydroxyvitamin D3 1-alpha-hydroxylase in the von Hansemann cells.

## 2 Case report

**Figure 1:**
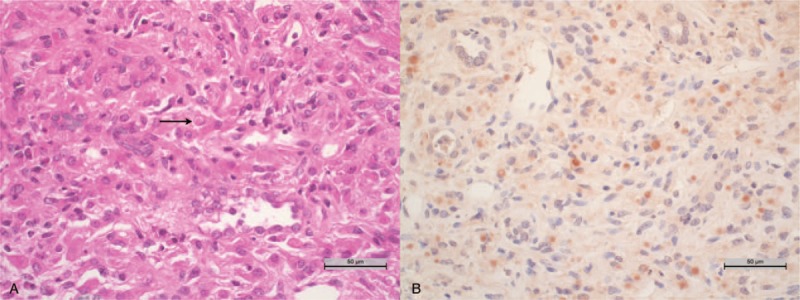
(A) Interstitial nephritis composed of macrophages with Michaelis–Gutmann bodies (arrow) revealing malakoplakia (hematoxylin and eosin; original magnification, x40). (B) Malakoplakia infiltrating cells with positive immunoreactivity for 25-hydroxyvitamin D_3_ 1-alpha-hydroxylase staining (original magnification, ×40).

A 65-year-old woman under tramadol was admitted in our institution because of fever and confusion after a 10 days course of ceftriaxone for an *Escherichia coli-*induced pyelonephritis. She was diagnosed with an acute renal injury, which had favored tramadol adverse effects since confusion receded after tramadol withdrawal. Regarding the acute renal injury, serum creatinine (SCr) was 182 μmol/L (eGFR_CKD-EPI_ 25 mL/min/1.73 m^2^), the urinary protein-to-creatinine ratio was 0.43 g/mmol (with a profile suggesting nonglomerular proteinuria), and urine microscopy revealed leukocyturia with *Enterococcus faecalis.* Imaging studies revealed medullary sponge kidneys without an obstructive cause for renal failure and bilateral nephromegaly (right kidney 165 mm and left kidney 155 mm). A renal biopsy was performed, revealing interstitial nephritis composed of macrophages with an abundant eosinophilic cytoplasm (von Hansemann cells) and Michaelis–Gutmann bodies in keeping with renal malakoplakia (Fig. 1A). The patient was given prolonged antibiotic therapy with cotrimoxazole. In addition, this patient with chronic kidney disease had 25-OH vitamin D insufficiency (56 nmol/L), for which she was prescribed oral cholecalciferol (100,000 IU per month) in spite of normal serum calcium (2.21 mmol/L) and parathyroid hormone (PTH) levels (38 ng/L). At the one-month follow-up after renal biopsy, her renal function had improved (SCr 137 μmol/L), as well as her 25-OH vitamin D serum levels and calcemia (67 nmol/L and 2.49 mmol/L respectively).

**Figure 2:**
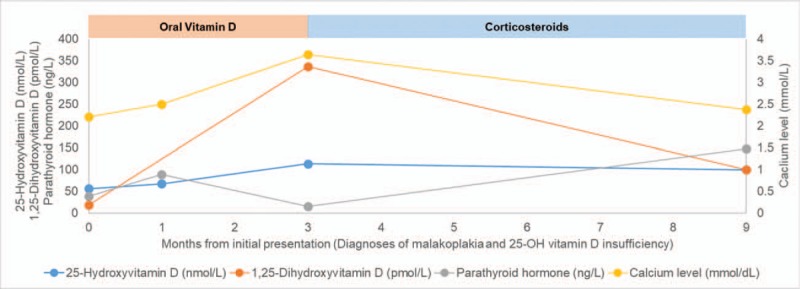
Values of serum 25-hydroxyvitamin D (ng/mL) (blue), 1,25-dihydroxyvitamin D (pg/mL) (red), parathyroid hormone (pg/mL) (green) and calcium (mg/dL) (purple) levels over time. Periods during which the patient received oral vitamin D (100,000 IU/month) and a steroid (0.5 mg/kg/day initially) are represented by red and blue rectangles, respectively.

Two months later, she was admitted to our unit for dehydration and hypercalcemia (3.64 mmol/L) with normal 25-OH vitamin D (113.1 nmol/L) and PTH (15 ng/L) levels and high 1,25-dihydroxyvitamin D levels (336 pmol/L), suggesting ectopic 25-hydroxyvitamin D_3_ 1-alpha-hydroxylase activity. Extensive investigations, comprising ^18^fluoro-deoxy-glucose positron emission tomography, bone marrow biopsy, thoracic computed tomography scanning, sputum examination for tuberculosis and a second renal biopsy, did not reveal a superimposed granulomatous disease. We suspected the malakoplakia cells to be responsible for the abnormal 25-hydroxyvitamin D_3_ 1-alpha-hydroxylase activity, and we performed immunohistochemistry for 25-hydroxyvitamin D_3_ 1-alpha-hydroxylase in slides from the renal biopsies (Fig. 1B). The test revealed ectopic expression of this enzyme by the infiltrating macrophages, whereas the infiltrating cells of a nonhypercalcemic sarcoidosis patient did not, although the tubular cells of this same patient did (data not shown). Cholecalciferol was stopped, the patient was rehydrated with intravenous physiological saline, and prednisone was initiated to decrease the enzyme activity. She was discharged with normal serum calcium (2.25 mmol/L). Six months later, serum calcium was 2.37 mmol/L, 25-hydroxyvitamin D was 99 nmol/L, 1,25-dihydroxyvitamin D was 100 pmol/L, and PTH was 147 ng/L (Fig. 2). Her renal function had stabilized at 202 μmol/L (eGFR_CKD-EPI_ 22 mL/min/1.73 m^2^). Unfortunately, the patient died 3 months later from cardiac failure caused by atrial fibrillation with normal calcium level.

## 3 Discussion

Malakoplakia is rare condition mostly affecting women over 40 years old. In most cases, it is localized in the urinary tract. It is identified by a pathological examination revealing macrophages with Michaelis–Gutmann bodies and Schiff-positive inclusions, which also stain for calcium and iron,^[1]^ corresponding to phagolysosomes containing residual bacteria remnants and, more especially, *E coli*.^[8–10]^ Indeed, malakoplakia is thought to be secondary to chronic local infections, favoring the decreased bactericidal activity displayed by the patients’ macrophages^[11,12]^ and accounting for the higher frequency of this disease in immunocompromised patients.^[13–15]^

Management of malakoplakia consists of the administration of intracellular penetrating antibiotics, such as quinolones and sulfamethoxazole-trimethoprim, associated with surgical resection or drainage,^[16]^ as well as withdrawal/reduction of immunosuppressive drugs when possible.^[17]^ In the present case, cotrimoxazole was chosen over ciprofloxacin because of the *Enterococcus faecalis* co-infection.

Clinical manifestations of malakoplakia are usually related to local infiltration, which induces pseudotumoral lesions, alters the architecture and function of the affected organ and may cause general symptoms such as asthenia and fever. In the present case, malakoplakia was also responsible for severe hypercalcemia through a 25-hydroxyvitamin D_3_ 1-alpha-hydroxylase enzymatic activity unmasked by vitamin D supplementation. We decided to give her oral vitamin D supplementation although she had insufficiency and not deficiency because of the expected benefit in patients with chronic kidney disease^[18]^ and the usual safety of this treatment. However, she unexpectedly developed severe hypercalcemia with high 1,25-dihydroxyvitamin D, suggesting a nonregulated activity of 25-hydroxyvitamin D_3_ 1-alpha-hydroxylase, which catabolizes the transformation of 25-OH vitamin D into the biologically active 1,25-dihydroxyvitamin D. After we excluded diseases commonly responsible for such ectopic activity, we suspected that the malakoplakia cells expressed the enzyme as in other granulomatous diseases such as sarcoidosis or mycobacterial infections,^[19]^ which was confirmed by immunostaining. Therefore, we prescribed high dose steroid to inhibit the ectopic 25-hydroxyvitamin D3 1-alpha-hydroxylase enzymatic activity as in granulomatous diseases induced hypercalcemia, although oral cholecalciferol withdrawal and malakoplakia treatment by antibiotic may have efficiently resolved this electrolyte disorder. However, long-term follow-up is unavailable since the patient, unfortunately, died from heart failure unrelated to renal disease or hypercalcemia.

Since it is the first case of malakoplakia causing severe hypercalcemia through a 25-hydroxyvitamin D_3_ 1-alpha-hydroxylase enzymatic activity it is not certain this may be generalized to all malakoplakia cases. It would necessitate performing immunohistochemistry for 25-hydroxyvitamin D_3_ 1-alpha-hydroxylase in slides from both renal and nonrenal malakoplakia cases.

Observations of concomitant sarcoidosis and malakoplakia in individual patients have caused some authors to propose that malakoplakia is a urinary bladder manifestation of sarcoidosis.^[20]^ However, it was later reported that in most of these cases, the diagnosis of sarcoidosis precedes that of malakoplakia by several years and that the patients are likely to receive, or explicitly receive, prednisone when malakoplakia is diagnosed. Therefore, malakoplakia is more likely a complication of the immunosuppressive therapy in these cases.^[3]^ Notably, our patient did not exhibit any signs of systemic sarcoidosis or granulomas in the renal biopsies.

In conclusion, this case demonstrates that malakoplakia cells may exhibit ectopic 25-hydroxyvitamin D_3_ 1-alpha-hydroxylase activity and cause severe hypercalcemia upon vitamin D supplementation. Therefore, such supplementation should not be given in malakoplakia patients without an actual deficiency and requires careful monitoring of serum calcium. Conversely, it can be assumed that hypercalcemia with low parathyroid hormone and high 1,25-dihydroxyvitamin D may reveal malakoplakia.

## Acknowledgements

The authors thank the HUPNVS of Assistance Publique - Hôpitaux de Paris for funding publications fees.

## Author contributions

**Conceptualization:** Jonathan Maurice Chemouny, Aurélie Sannier, Eric Daugas.

**Formal analysis:** Jonathan Maurice Chemouny, Eric Daugas.

**Investigation:** Jonathan Maurice Chemouny, Aurélie Sannier, Guillaume Hanouna, Laure Champion, François Vrtovsnik, Eric Daugas.

**Methodology:** Jonathan Maurice Chemouny, Eric Daugas.

**Supervision:** Eric Daugas.

**Validation:** Aurélie Sannier, Guillaume Hanouna, Laure Champion, Eric Daugas.

**Visualization:** Jonathan Maurice Chemouny, Guillaume Hanouna, Eric Daugas.

**Writing – original draft:** Jonathan Maurice Chemouny, Eric Daugas.

**Writing – review & editing:** Jonathan Maurice Chemouny, Aurélie Sannier, Guillaume Hanouna, Laure Champion, François Vrtovsnik, Eric Daugas.

Jonathan Maurice Chemouny: 0000-0001-6309-3986
